# Cost-effectiveness of childhood pneumococcal vaccination program in Ethiopia: results from a quasi-experimental evaluation

**DOI:** 10.1186/s12889-019-7423-8

**Published:** 2019-08-09

**Authors:** Tayue Tateke Kebede, Mikael Svensson, Adamu Addissie, Birger Trollfors, Rune Andersson

**Affiliations:** 1 Department of Public Health and Community Medicine, Institute of Medicine, Sahlgrenska Academy, University of Gothenburg, Gothenburg, Sweden; 2 Health Metrics Unit, Sahlgrenska Academy, University of Gothenburg, Gothenburg, Sweden; 3 Department of Preventive Medicine, School of Public Health, Addis Ababa University, Addis Ababa, Ethiopia; 4 Department of Paediatrics, Sahlgrenska University Hospital, Gothenburg, Sweden; 5 Department of Infectious Diseases, Institute of Biomedicine, Sahlgrenska Academy, University of Gothenburg, Gothenburg, Sweden

**Keywords:** Pneumococcal vaccination, Childhood respiratory infection, Cost-effectiveness

## Abstract

**Background:**

Ethiopia was among the 15 countries that, together accounted for 64% of the world’s severe episodes of pneumonia among children below the age of 5 in 2011. To reduce this burden, the 10-valent pneumococcal conjugate vaccine (PCV 10) was introduced into the general childhood national immunization program in Ethiopia in 2011. However, there is little evidence on its cost-effectiveness, and the aim of this study was to estimate the cost-effectiveness of the introduction of PCV 10 vaccination in the Ethiopian setting.

**Methods:**

The cost-effectiveness analysis was carried out based on a quasi-experimental evaluation of implementing PCV 10 at the Butajira rural health program site in Ethiopia. The intervention and the control groups consisted 876 and 1010 children, respectively. Using data from program site’s surveillance system database as a framework, health outcome and vaccination data were collected from medical records, immunization registration books and reports. Disability- Adjusted Life Year (DALY) was a main health outcome metric complimented by incidence of acute lower respiratory infection/1000-person years. Vaccination and treatment costs were collected by document review and cross-sectional household survey.

**Results:**

In the intervention cohort, 626 of 876 (71.5%) children received PCV 10 vaccination. Until the first year of life, the incidence of acute lower respiratory infection was higher in the intervention group. After the first year of life, the incidence rate was 35.2 per 1000-person years in the intervention group compared to 60.4 per 1000-person years in the control group. The incremental cost-effectiveness ratio (ICER) per averted DALY for the intervention group during the total follow-up period was (2013 US$) 394.3 (undiscounted) and 413.8 (discounted). The ICER per averted DALY excluding the first year of life was (2013 US$) 225 (undiscounted) and 292.7 (discounted).

**Conclusion:**

Compared to the WHO’s suggested cost-effectiveness threshold value, the results indicate that the general childhood PCV 10 vaccination was a cost-effective intervention in the Butajira rural health program site.

**Electronic supplementary material:**

The online version of this article (10.1186/s12889-019-7423-8) contains supplementary material, which is available to authorized users.

## Background

Child health has been a major public health priority during the past few decades at global and regional levels. Child health improvements represented a major part of the 1990–2015 millennium development goals (MDGs), and the international and local communities committed to reducing mortality in children under-five by two thirds [1]. Now the MDGs implementation period is over and the effect of the interventions and commitments can be measured, and lessons can be drawn. Globally, the mortality rate in children under five decreased by 52.4%, from 87.1 per 1000 live births in 1990 to 41.4 per 1000 live births in 2015 [2]. Though the progress was encouraging, the goal was not achieved. In addition, progress was not uniform between and within countries [2, 3]. In sub-Saharan Africa, for instance, most of the countries could not meet the target set by the MDG. The aggregated under-five mortality rate in the region was 98 deaths per 1000 live births in 2012, which was far above the MDG 2015 target of 59/1000 live births. Furthermore, in 2013 the region contributed to approximately half of the under-five deaths in the world [4, 5].

In 2013, pneumonia, diarrhoea, and malaria were the leading causes of under-five death globally with disproportionate burden on low-income countries. Furthermore, in the above-mentioned years, the number of children who died from pneumonia was nearly as high as the sum of children who died from diarrhoea and malaria combined [6]. Despite a reduction in pneumonia-related deaths by 44% between 2000 and 2013, pneumonia still accounted for 15% of the total under-five deaths in 2013 [7].

Ethiopia was one of the 15 countries that contributed to 65% of world’s total episodes of pneumonia and to 64% of severe episodes of pneumonia in 2011 [8]. Furthermore, Ethiopia had globally the fifth highest mortality rate from pneumonia and diarrhoea with 53,000 deaths in 2013 [7]. Lower respiratory infections in general, and pneumonia specifically, were the leading causes of under-five deaths [9, 10].

Vaccination has been shown to be an effective preventive intervention for pneumonia and two major versions of conjugate vaccine, 10-valent (PCV 10) and 13-valent (PCV 13) pneumococcal conjugate vaccines, have been approved and included in routine childhood immunization programs across the world [11–13]. Ethiopia introduced PCV 10 into the childhood immunization program in November 2011 with the support of Gavi, the Vaccine Alliance [14, 15]. In the three-dose schedule of the national vaccination program, children are given the vaccine at their 6, 10 and 14 weeks of life. However, at the time of its introduction in Ethiopia, all children below one year of age were supposed to be vaccinated as part of a catch-up campaign.

Most studies on the cost-effectiveness of PCV 10 across the world have shown this to be a cost-effective intervention [16–22]. However, there are substantial differences between studies in the considered comparator, outcome measures and effectiveness estimates [23–25]. Some studies have questioned the cost-effectiveness of PCV 10, e.g. in Thailand and Croatia [26, 27]. In Ethiopia, with the exception of some recent reports of extended cost-effectiveness analysis, which were mainly based on international estimates, there is limited evidence as to the cost-effectiveness of universal PCV 10 vaccinations [28, 29].

Ethiopia lacked a national vital registration system and population-based longitudinal demographic and health information were limited to a few university-based health and demographic surveillance systems. Thus, we used the Butajira rural health program (BHRP), which is one of the university-based health and demographic surveillance systems, as a framework to measure the cost-effectiveness of PCV 10 vaccination. Addressing the knowledge gap regarding the cost effectiveness of the vaccination will help policy makers and the health care providers to make evidence-based decisions and reliable prioritization in child health cares. Thus, this study aimed to measure the cost effectiveness of PCV 10 vaccination in the program site.

## Methods

### Study population and data collection

The evaluation compares a universal vaccination program (in which all age eligible children are given full entitlement of getting PCV 10 vaccination without payment) with no universal vaccination program (i.e. still may have self-selection into vaccination). The study population used to collect data on costs and health outcomes was children born in the BHRP site. The site was established in 1986 in a collaboration between Addis Ababa University, Ethiopia and Umeå University, Sweden. It comprised one urban and nine rural kebeles (the smallest administrative unit in Ethiopia) from Meskan and Mareko districts of Gurage and Silte zones [30, 31]. Geographically defined populations of individuals, households and residential units were followed as an open cohort in the selected kebeles. Demographic and health related events, mainly pregnancy (observation and outcome), death, in and out migration and marital status changes were the major units of updates that were taken in every three months in the surveillance system. Causes of death were determined by verbal autopsy (VA) method using international classification of diseases (ICD-10) codes and titles. Then the ICD-10 codes and titles subsequently converted into VA codes and titles [31]. The sites population grew from 28,000 in 1987 to over 70,000 in 2011 [32].

Children in the intervention group (i.e. within the time period of universal vaccination) were born between September 1, 2011 and August 31, 2012. Those in the historical control group (i.e. within the time period of no universal vaccination) were children born between September 1, 2009 to August 31, 2010. The control group were chosen over this period to ensure that they were not included in the catch-up programs.

Children who died or who had emigrated before the age of two months or who had immigrated to the study area after their first year of life were not included in the study. Data were collected from November 25, 2015 until January 6, 2016. Children in both the intervention and control cohorts were monitored retrospectively to an age of three years and three months of life (final data collection took place in January 2016), as this was the maximum time we could consider for children born in late 2012. list of the study participants was extracted from the HRS2 (household registration system 2- longitudinal data management software of the program) into excel and a format was prepared to track their history in relation to the study objective. Their full names, residence kebeles, sub-kebeles and dates of birth were considered as an identifier and units of integration for the HDSS data with health system data. Using the list, document number of the children were identified from the HMIS (health management information system) and/or registration book of hospitals and health centers. Vaccination data were collected by health extension workers and nurses. Morbidity data were collected by nurses. Household survey data were collected by BRHP data collectors using pretested questionnaire (Additional file 1). All data collectors were trained on procedures of the data collection and the objectives of the study.

### Sample demographics

The two cohorts of children, 876 in the intervention group and 1010 in the control group, were homogenous in terms of residence and sex distribution. However, there were statistically significant differences regarding place of birth and birth attendant (see Table 1). The births of 253 (28.9%) children in the intervention group and 138 (13.7%) children in the control group were attended by health professionals. Mothers to the children in the intervention group had a lower mean pregnancy rate and parity than their counterparts in the control group, 3.8 ± 2.4 versus 4.2 ± 2.5 (*p* = 0.004) and 3.9 ± 2.4 versus 4.3 ± 2.5 (*p* = 0.001), respectively. From a total of ten kebeles identified in the program site, two were excluded due to missing data and inconsistent values in the HDSS database. A further eleven children were excluded because they had sepsis without respiratory symptoms where the focus of the infection was not possible to define.

**Table 1:** Background information relating to intervention (Sep, 2011 – Aug, 2012) and control (Sep, 2009 – Aug, 2010) groups at the Butajira rural health program site

Variables	Intervention Group (*n* = 876)	Control Group (*n* = 1010)	Total (*n* = 1886)	*P*-value
	Freq. (%)	Freq. (%)	Freq. (%)	
Residence				
Rural	607 (69.3)	726 (71.9)	1333 (70.7)	0.218
Urban	269 (30.7)	284 (28.1)	553 (29.3)	
Sex				
Male	449 (51.3)	503 (49.8)	952 (50.5)	0.529
Female	427 (48.7)	507 (50.2)	934 (49.5)	
Place of birth				
Home	623 (71.3)	852 (84.9)	1475 (78.5)	< 0.001
Health center	75 (8.6)	26 (2.6)	101 (5.4)	
Hospital	167 (19.1)	112 (11.2)	279 (14.9)	
Others	9 (1.0)	14 (1.4)	23 (1.2)	
Unknown*	2 (0.2)	6 (0.6)	8 (0.4)	
Birth attended by:				
Neighbors	424 (48.4)	539 (53.4)	963 (51.1)	< 0.001
Health professionals	253 (28.9)	138 (13.7)	391 (20.7)	
TBA	75 (8.6)	123 (12.2)	198 (10.5)	
Relatives	71 (8.1)	133 (13.2)	204 (10.8)	
TTBA	46 (5.3)	56 (5.5)	102 (5.4)	
Have no assistant	3 (0.3)	7 (0.7)	10 (0.5)	
Others	4 (0.5)	14 (1.4)	18 (1.0)	
Number of pregnancies of their mothers				
1	166 (18.9)	153 (15.1)	319 (16.9)	< 0.001
2–4	321 (36.6)	392 (38.8)	713 (37.8)	
≥ 5	267 (30.5)	390 (38.6)	657 (34.8)	
Unknown *	122 (13.9)	75 (74)	197 (10.4)	
Parity of their mothers				
1	164 (18.7)	146 (14.5)	310 (16.4)	< 0.001
2–4	324 (37.0)	389 (38.5)	713 (37.8)	
≥ 5	266 (30.4)	400 (39.6)	666 (35.3)	
Unknown *	122 (13.9)	75 (7.4)	197 (10.4)	

Notes: TBA- traditional birth attendant, TTBA- trained traditional birth attendant, Freq. – frequency

* = data were not found in HDSS data system

### Economic costs

Costs were estimated by taking into account both the resources consumed for the vaccination and associated healthcare treatments. The former costs were estimated based on the data in the Gavi-approved proposal of the country to introduce PCV vaccination [33, 34] with an assumed wastage rate of 10% for the vaccine. Healthcare treatment estimations included medical and non-medical costs related to both outpatient and inpatient care. Non-medical costs such as costs for transportation, meals, accommodation, productivity loss for parents and caregivers, were estimated from cross-sectional household surveys. The medical cost of the infections was also determined from the cross-sectional household survey by asking interviewee about their recent medical expenses to treat their infected children. Since out-of-pocket payment covered 78.1% of the total direct medical cost for patients who get medical care from public providers, 21.9% of the estimated cost was added to determine the total mean direct medical cost [35]. Data on drug costs were extracted from public pharmacies.

The household survey sampled 134 of the 375 total households with the identified sick children in the current study. These were chosen by cluster-randomization (with the ten kebeles as the clusters) with each household head and/or mother interviewed regarding their recent expenses for treating their sick children. Then the costs were converted to 2013 US$ value. Pretested interview guide was used to interview the respondents.

We took 2013 as a base year for costs with the assumption that it is a reasonable time to capture representative mean treatment cost for both cohorts and mean vaccination cost; since the vaccination was introduced in the late 2012. Summary of the mean cost (per cost item) for three doses of PCV 10 is shown in Table 2.

**Table 2:** Parameter assumptions and calculated values regarding costs, health outcomes and discounting

Parameters	Values	References
Costs (2013 US$)		
Mean vaccine unit price per dose	6.5	[33]
Auto-disposable syringes unit price	0.069	[33]
Safety boxes unit price	0.94	[33]
Introduction, initial costs per child	0.17	[33]
Administration costs per child	0.25	[33]
Cold chain equipment and maintenance cost per child	0.02	[33]
Other costs per child	1.99	[33]
Freight cost for vaccine and devices as % of vaccine values	10%	[33]
Mean vaccination cost per child	26.88	[33]
Mean total indirect medical cost of infections		
Mean transportation cost	5.8	Survey^ϯ^
Mean meals cost	9.5	Survey^ϯ^
Mean accommodation cost	1.8	Survey^ϯ^
Mean total direct medical cost of infections		
Mean inpatient cost	1.4	Survey^ϯ^
Mean diagnosis cost	2.3	Survey^ϯ^
Mean home care cost	0.3	Survey^ϯ^
Mean drugs cost	5.9	Survey^ϯ^
Mean lost productivity cost per child	30.8	Survey^ϯ^
Disability and age weights		
Pneumonia*	0.28	[36, 37]
Sepsis	0.28	[16, 37]
Acute otitis media	0.334	[16]
Meningitis	0.62	[34]
Age weights	0.04	[38]
Mean duration of illness (in days)		
Pneumonia, Sepsis and Acute otitis media	6	
Meningitis	10	[34]
General parameters		
Life expectancy (years)	65	[39]
Discount rate	3%	[38]

* we used the value of pneumonia disability weight for ALRI

Ϯ the values are estimated based on the household survey that was performed as part of this study.

### Health outcomes data

The primary health outcome metric used was Disability-Adjusted Life Years (DALYs). It was estimated using the excel formula suggested by Fox-Rushby and Hanson [40]. The potential illnesses considered to estimate DALYs included all-cause acute lower respiratory infections (ALRI), all-cause meningitis (AM), acute otitis media (AOM), sepsis, and death. Disability weights and duration of illnesses are shown in Table 2. Estimates on the incidence of AM, AOM, ALRI and sepsis were based on the quasi-experimental evaluation comparing the intervention group to the historical control group. Prevented incidence of ALRI/1000-person years was also an additional outcome metric in the current study. Data on morbidity were collected from medical records at hospitals and health centers in the catchment area of the surveillance site. It was done by nurses (BSc nurses), who were working at respective hospitals and health centers. Checklist was used to collect the data and categorization of the cases to the infections was done based on the diagnosis of the hospitals and the health centers. Vaccination status of the children was collected from immunization registration books of hospitals, health centers and health posts. Demographic and mortality data were extracted from the surveillance system database. To calculate Life-years lost where children died, we assumed the average life expectancy to be 65 years (year 2014).

### Cost-effectiveness analysis

A decision analytic model, programmed in Microsoft excel considering previous studies [25, 41], was used to estimate the cost effectiveness of universal PCV 10 vaccination compared to no universal PCV 10 vaccination. Incremental cost-effectiveness ratios (ICERs) were estimated from a societal perspective in terms of the cost per averted DALY and the cost per prevented incidence of ALRI/1000-person years. The results were generated both with and without discounting. The discount rate was set at 3% for the costs and the outcomes, in line with recommendations (e.g. the second US panel on cost-effectiveness) [42]. The cost per averted DALY was compared to the threshold levels suggested by the Commission for Macroeconomics and Health as three times the per-capita gross domestic product (GDP) of the country, which was US$ 505 in 2013 [43, 44]. The GDP-based threshold has been criticized for its limitations and lack of empirical foundation. Nonetheless, it is the commonly-used policy-relevant comparative standard [45]. A one-way deterministic sensitivity analysis was used to determine parameter uncertainties.

## Results

### Vaccination history

In the intervention cohort, 626 (71.5%) children received at least one dose of PCV 10. Among them, 623 (99.5%) received 3 doses of the vaccine. Regarding the other children, 35 (4.0%) received no vaccine and the vaccination status of 215 (24.5%) children was unknown. Residentially, 483 (77.2%) of the vaccinated children were rural dwellers and 143 (22.8%) urban dwellers; the difference was statistically significant (*p* < 0.001). Girls and boys had similar vaccination status with respective percentages of 73.3% versus 69.9% for vaccinated status, 3.0% versus 4.7% for unvaccinated status and 23.7% versus 25.4% for unknown vaccination status (*p* = 0.28). The majority (54.6%) of children received PCV vaccine at health posts followed by hospitals (11.5%) and health centers (5.4%). In the subsequent sections, we focus on the intention-to-treat (ITT) results, i.e. comparing the full intervention cohort to the historical control group (irrespective of vaccination status).

### Lower respiratory infections

ALRI was the leading cause of healthcare visits in the current study with a total of 204 episodes (range: 1–4 per individual) and 211 (range: 1–7 per individual) in the intervention and the control groups, respectively. The infection proportion were similar in the two groups, with 17.8%, in the intervention group compared to 16.3% in the control group, the difference not being statistically significant (*p* = 0.43). In the intervention cohort, ALRI was still the leading infection irrespective of vaccination status. The infection proportion in the vaccinated sub-group was 16.1% whereas, for children with a vaccination status of unknown and unvaccinated, the percentages were 22.8 and 14.5%, respectively.

In the intervention group, the infection proportion was 24.5% in urban and 14.7% in rural areas (*p* = 0.001). Likewise, in the control group the corresponding figure was 22.2 and 14.0% (*p* = 0.002). The differences in the infection among urban children in the intervention and control groups were not statistically significant.

### Incidence of ALRI and the survival rates for those children not contracting the infection

In the current study, the incidence rate of ALRI decreased as the children in the intervention group grew older. For the whole period of the study, the incidence rate in the intervention group was 88.5 per 1000 person-years compared with 75.2 per 1000 person-years in the control group. Looking at the sub-set of data after the children’s first birthday, the incidence rate was 56.4 in the intervention group compared to 60.4 per 1000 person-years in the control group (Table 3).

**Table 3:** Incidence rate for infections per 1000 person-years and number of deaths at Butajira rural health program site during three years and three months from inclusion in the study in the intervention group, the vaccinated sub-set of the intervention group (Sep. 2011 – Aug. 2012), and control group (Sep 2009 – Aug. 2010)

Infection	Intervention group (*n* = 876)		Control group (*n* = 1010)
	All (876)	Vaccinated sub-group (626)	
Acute lower respiratory infections			
≤ 6 months	138.9	124.5	94.0
7–12 months	76.9	84.8	54.2
**≥**12 months	56.4	30.6	60.4
**≥** 2 years	26.1	22.8	29.6
For the total period	88.5	82.1	75.2
Acute otitis media (for all)	7.1	5.0	6.2
Sepsis (for all)	4.3	3.7	0.4
Death	2	2	6

**Fig. 1:**
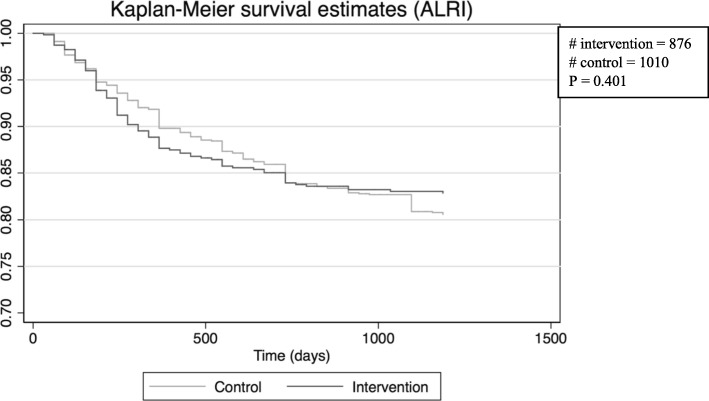
Survival curves of first time acute lower respiratory infection (ALRI) at Butajira rural health program site, for three years and three months from inclusion in the study in the intervention group (Sept. 2011 – Aug. 2012) and control group (Sept. 2009 – Aug. 2010)

**Fig. 2:**
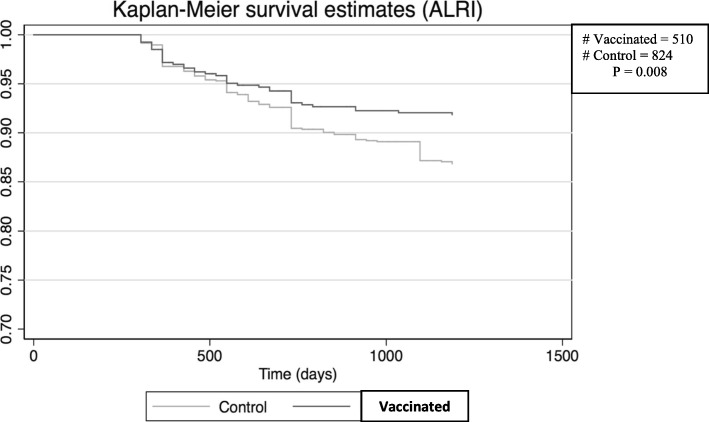
Survival curves of first time acute lower respiratory infection (ALRI) at Butajira rural health program site, after one year of life in the vaccinated sub-groups (Sept. 2011 – Aug. 2012) compared to control group (Sept. 2009 – Aug. 2010)

The age at which children contracted ALRI tended to be lower in the intervention group compared to the control group. The mean age for the first infection was 11.9 ± 9.0 and 15.0 ± 10.5 months for the intervention and control groups, respectively. Until the first year of life, children in the intervention group tended to have a lower ALRI free survival compared to the control group (*p* = 0.401) (Fig. 1). However, after their first birthday, children in the intervention group tended to have a higher ALRI free survival than their counterparts in the control group (*p* = 0.207). After their second year, they had statistically significant higher ALRI free survival (*p* = 0.01) similar with children in the vaccinated sub-group after their first birthday (*p* = 0.008) (Fig. 2). For meningitis, AOM and sepsis, most of the children contracted the infection after 6 months of life. Only one case of meningitis was reported from the intervention group.

### Hospital admission, mortality and cohort dynamics

Hospital admissions due to severe ALRI were more frequent in the intervention group compared to the control group, 10.2% vs 3.4%, (*p* = 0.01). The majority (82%) of admissions in the vaccinated sub-group in the intervention group were among children below the age of one year of age.

There were 13 (2.1%) and 35 (3.5%) deaths in the intervention and control groups, respectively. Among those who died, 23% in the intervention and 8% in the control groups had history of healthcare visits due to ALRI or meningitis. Furthermore, ALRI was the cause of death for two children in the vaccinated and for six children in the control group. On the other hand, 140 (14.2%) and 266 (22.7%) children left the surveillance site before the end of the observation in the intervention and control groups, respectively. Among them, 16.4% in the intervention and 16.2% in the control group got ALRI infections as shown in Table 4.

**Table 4:** History of infections among children who left the study areas before completion of observation period during three years and three months from inclusion in the study in the intervention group (*n* = 140; Sep. 2011 – Aug 2012) and control group (*n* = 266; Sep. 2009 – Aug. 2010) at Butajira rural health program site

Infection	Freq. (%)	Freq. (%)
Acute lower respiratory infection	23 (16.4)	43 (16.2)
Acute otitis media	5 (3.6)	5 (1.9)
Sepsis	3 (2.1)	4 (1.5)
Meningitis	0	0

### Vaccination program and treatment cost

At the introduction year of the vaccination, the total program cost was US$ 26.88 per child for the three doses of PCV 10. The vaccine, the auto-disposable syringe and the safety box together cost US$ 26.44. Other vaccine introduction activities, i.e. training, social mobilization, cold chain equipment procurement and maintenance, transportation and other items amounted to a cost of US$ 0.44 per child.

The mean cost of treating children for an episode of the considered infections in the current study was 513.7 ± 487.5 birr (local currency) or 26.9 US$. The mean direct medical cost was birr 186.4 ± 153.9 (US$ 9.8) and the mean indirect medical cost was birr 327.3 ± 333.6 (US$ 17.1). Likewise, the mean cost of lost productivity was 588.6 birr (US$ 30.8). The mean incremental cost of PCV 10 vaccination was US$ 26.6 for the children over the age of one year.

### Cost-effectiveness of the intervention

The mean DALYs (per child) were 0.08 and 0.20 in the intervention and control group, respectively. This implies 0.12 averted DALYs in the intervention group. With an incremental cost of 33.3 US$, this translates to an ICER of 281.4 (discounted) and 268 (undiscounted) US$ per averted DALY in the total follow up period, Table 5.

**Table 5:** Incremental cost-effectiveness of 10- valent pneumococcal conjugate vaccine in terms of averted DALYs during three years follow-up from inclusion in the study in the intervention group, vaccinated sub-group (Sep. 2011 – Aug. 2012) and control group (Sep. 2009 – Aug. 2010) at Butajira rural health program site

Health outcomes	Cost*	Incremental cost	Effectiveness	Incremental effectiveness	Incremental cost- effectiveness ratio (ICER)	
					No discounting	Discounting 3%
DALYs						
< 1 year of age						
Intervention	36.7	30.2	0.08	−0.01	Dominated	Dominated
Vaccinated	36.2	29.7	0.11	−0.04	Dominated	Dominated
Control	6.5		0.07			
> 1 year of age						
Intervention	36.3	30.0	0.0001	0.14	219.4	227.9
Vaccinated	32.9	26.6	0.0001	0.13	194.2	273.9
Control	6.3		0.1338			
> 2 years of age						
Intervention	31.0	29.2	0.00005	0.103	283.7	318.6
Vaccinated	29.1	27.2	0.00003	0.1009	264.9	282.6
Control	1.86		0.103			
Total follow-up period						
Intervention	46.1	33.3	0.08	0.12	268.0	281.4
Vaccinated	42.2	29.3	0.11	0.10	304.7	342.0
Control	12.8		0.20			

* N.B: the costs for averted DALYs and ALRI incidences are not the same due to the difference in considered costs. For DALYs, all costs in relation to all infections and vaccination were considered. In the case of ALRI, only costs related to ALRI and vaccination were considered

The mean DALYs, after the twelfth month of age, was 0.0001 for the entire intervention group (including the vaccinated, in particular) versus 0.1338 for the controls, yielding 0.14 averted DALYs. In this study, the ICER translates to 227,9 (discounted) and 219.4 (undiscounted) US$ per DALY averted, respectively (Table 5). Likewise, the ICER of averting an ALRI incidence per 1000-person years after the twelfth month and its discounted value for the intervention group were US$ 6.7 and 7.5, respectively (Table 6). For the full-time period, the intervention was dominated in terms of reduced ALRI incidence, since the intervention group had higher cost and worse health outcomes. This implies that the averted DALYs in the intervention group (as reported above) is a consequence of the fewer deaths, where (considering the young age of the children), each death increases the lost life-years.

**Table 6:** Incremental cost-effectiveness of 10- valent pneumococcal conjugate vaccine in terms of prevented ALRI incidence/1000-person years during three years follow-up from inclusion in the study in the intervention group, vaccinated sub-group (Sep. 2011 – Aug. 2012) and control group (Sep. 2009 – Aug. 2010) at Butajira rural health program site

Health outcomes	Cost*	Incremental cost	Effectiveness	Incremental effectiveness	Incremental cost- effectiveness ratio (ICER)	
					No discounting	Discounting 3%
Incidence of ALRI/1000-person years						
< 1 year of age						
Intervention	36.35	29.7	154.6	−46.6	Dominated	Dominated
Vaccinated	34.0	27.39	154.5	−46.5	Dominated	Dominated
Control	6.6		108.0			
> 1 year of age						
Intervention	29.3	26.4	56.4	3.96	6.7	7.5
Vaccinated	28.7	25.9	30.6	29.8	0.9	1.0
Control	2.83		60.4			
> 2 years of age						
Intervention	28.3	25.8	26.1	3.5	7.3	9.7
Vaccinated	28.7	26.2	22.8	6.8	0.26	3.1
Control	2.5		29.6			
Total follow-up period	27					
Intervention	39.7	28.1	88.5	−13.3	Dominated	Dominated
Vaccinated	39.5	.9	82.1	−7.0	Dominated	Dominated
Control	11.6		75.2			

### Sensitivity analysis

One-way sensitivity analysis was done by varying the costs and infection rates by ±20% to evaluate how robust the results are to changes in parameter values. The ICER (undiscounted) was most sensitive to ±20% change in vaccination cost in the intervention group with a change in ICER (undiscounted) ranging from US$ 224.7 to 311.2 per averted DALY (Table 7). ICER (undiscounted) remained within 10% of the base case after ±20% changes in most parameters for the treatment and control groups.

**Table 7:** One-way sensitive analysis results at Butajira rural health program site

Parameter	Group	Variation (%)	ICER
Base case			268.0
Treatment cost	Intervention	−20	253.5
		20	282.4
	Control	−20	277.6
		20	258.3
Vaccination cost	Intervention	−20	224.7
		20	311.2
Lost productivity cost	Intervention	−20	251.4
		20	284.5
	Control	−20	279.0
		20	256.9
Total infection rate	Intervention	−20	236.7
		20	299.2
	Control	−20	288.6
		20	247.3

## Discussion

This study estimated the cost-effectiveness of the PCV 10 vaccination in the Butajira rural health program site in Ethiopia. The study design was based on comparing the first cohort of children born after the general vaccination was introduced with the cohort of children born the year before the introduction of the vaccination. The children were followed retrospectively from their second month of life to an age of three years and three months by the surveillance system and using medical records to determine the incidence of all-cause acute lower respiratory infections, meningitis, otitis media, sepsis and death.

The (discounted) cost per averted DALY was 281 (2013 US$), which is lower than the Ethiopian GDP per capita of 505 US$ (2013). This indicates that the intervention was cost-effective for the whole follow-up period based on the standard threshold value for cost-effective interventions suggested by the WHO [46]. Looking at the data after the first year of age, the (undiscounted) ICER (2013 US$) per averted DALY and ALRI incidences prevented was 219.4 and 6.7, respectively. The cost-effectiveness estimates are higher compared to the estimates for GAVI eligible countries of US$ 112 per averted DALY among under five children and US$ 77 in countries with under five mortality rate of 100–149 per 1000 live births [47]. It is also higher than the estimate based on data from Kenya, which was 2010 US$ 59 per averted DALY. However, it is lower than Gambian estimates of 2005 US$ 670 per averted DALY [48].

Focusing on data during the children’s first year of age, the vaccination was not cost-effective due to the higher incidence rate of ALRI in the intervention group. The highest rate of ALRI in the full intervention and the sub-group of vaccinated children, 138.9 and 124.5 per 1000 person-year respectively, was recorded in children under the age of six months. It decreased to 56.4 and 30.6 per 1000 person-years, respectively, after the first year of age, compared to a reduction from 94.0 to 60.4 per 1000 person-year in the control cohort. The reduction was 59% in the intervention group and 75% in the vaccinated sub-group compared to 36% in the control group. This is greater than the reduction of invasive pneumococcal disease incidence in the Gambian study, in which the incidence decreased by 55% (from 253 cases per 100,000 population to 113 cases per 100,000) in children aged 2–23 months [49]. Though that study used a health and demographic surveillance system (HDSS) similar to ours, a prospective rather than a retrospective study design was used and took wider age group than ours. In addition, the considered health outcome in our case was ALRI rather than invasive pneumococcal diseases only.

During the whole follow-up period, the intervention group had a higher incidence rate (88.5 per 1000-person year) than the control group (75.2 per 1000 person-year). This was partly due to the highest incidence rate (102.5 per 1000 person-year) among the unknown vaccination status sub-group in the intervention cohort. However, the mortality rate was lower in the intervention group (2.1% vs 3.5%), which implied that the total number of DALYs was lower in the intervention group compared to the control group (0.08 vs 0.20 per child). We don’t have data on why the ALRI was much higher among the unknown vaccination status sub-group of children in the current study. But it is likely that they had lower level of vaccination and parents with lower education and lower socioeconomic conditions including more frequent cooking on open fire with indoor air pollution.

Furthermore, children in the intervention group might have been utilizing healthcare more effectively as they had a higher proportion of institutional deliveries and health professional birth attendances. The highest incidence rate in the vaccinated sub-group was during the first year of life. This could be due to several reasons, e.g. since the follow-up period started immediately after the introduction of the vaccination, there was a catch-up campaign to allow children to receive the first dose of the vaccine before their first birthday. Hence, although they belonged to the intervention group, some of them received the vaccination late and the effect of the vaccine may therefore have been delayed. Some of the children might not get age-appropriate dose despite the vaccination schedule states that the last dose should be given at 14 weeks of age. This is in line with a recent study in Sweden that also reported a higher incidence of invasive pneumococcal diseases (IPD) among younger children post-PCV introduction [50]. Another study also pointed out a higher probability of being infected by non-vaccine serotypes between 2 and 11 months of age [49]. Finally, since it was not possible to define the specific cause of infection, the higher incidence of infection might be due to viral or other bacterial causes. In this case, respiratory syncytial virus (RSV) virus might have contributed, since it is a common cause of pneumonia in the first year of life [51, 52].

However, in the current study, the incidence of ALRI in the first year of life was lower than reported from a study in South Africa, where the corresponding figures for two months to one year was 0.29 episodes per child-year after the introduction of PCV 10 [53]. Although different methods were used, we suggest the difference is due to under-reporting in our study since we used medical records from healthcare facilities, and also because of the low healthcare utilization for respiratory infections [54].

Children who were living in urban areas had a higher rate of ALRI than rural children in both the intervention and control groups. This could potentially be explained by the fact that children in urban areas usually have more frequent human contacts, thus possibly resulting in higher levels of transmission in respect of the infection [55, 56]. On the other hand, since migration is greater in urban areas, newcomers to the towns might have less access to healthcare. Furthermore, among the intervention cohort, the vaccination rate was higher in rural areas than urban areas, 79.9% versus 53.2%. This difference might be due to the implementation of the health extension program in rural parts of the country, including the rural part of the current study setting [57].

Complimenting DALYs with incidence of ALRI/1000-person years as an outcome metrics added strength to the current study by providing better illustration of the effect of the vaccination. Considering the former metrics in the framework of 65 years life expectancy might not be adequate to inform on the value of the vaccination [58, 59]. For instance, for the full follow-up period, the intervention was dominated in terms of reduced ALRI incidence but cost effective in terms of averted DALYs. That was so due to the higher contribution of lost life years in DALYs calculation resulted from fewer more deaths in the control group than in the intervention group. So, it is important to combine such measures to get a better evidence of the effect in a country like Ethiopia where how long the effect of the vaccine lasts is not yet determined.

It is worthy of note that the study has a number of limitations**.** Only the public healthcare part of the health system was considered in order to determine the cost-effectiveness of the vaccination. Though generally, vaccination was provided at the public healthcare institutions, it is not uncommon in Ethiopia for people to visit private healthcare providers when they fall ill. Therefore, the exclusion of the private healthcare sector might introduce bias. In addition, patients may bypass the usual referral system by attending hospitals or health centers outside the catchment area of the local healthcare institutions. This could reduce the geographic precision of healthcare costs and healthcare utilization rates and the effectiveness results. Microbiological testing was not done and in consequence specific causes of the infections were not identified. Our study design with a historical control might also introduce biases as it has a limitation in terms of identifying casual effects of vaccination in the case of infectious diseases, where there is considerable seasonal variation in the epidemiology of the infections. Recall bias might also affect our household survey results. Finally, any indirect effects of the vaccination, either the herd immunity or the serotype replacement, were not considered in the current study. Thus, further investigation with robust methodology considering serotype prevalence and drug resistance is warranted to discern the long-term impacts of the vaccination in the Ethiopian setting.

## Conclusion

The 10- valent pneumococcal vaccination was a cost-effective intervention in terms of averted DALYs of acute lower respiratory infections in the intervention group at Butajira rural health program site. It is mainly due to lower acute lower respiratory infection related mortality in the intervention group than in the control group.

## Additional file

Additional file 1: Household survey questionnaire. (DOCX 17 kb)

## Data Availability

The datasets used and/or analysed during the current study are available from the corresponding author on reasonable request.

## Abbreviations

ALRI: acute lower respiratory infections
AM: all-cause meningitis
AOM: acute otitis media
BRHP: Butajira rural health program
BSc: Bachelor of science
DALY: Disability- Adjusted Life Year
Gavi: the Vaccine Alliance
GDP: gross domestic product
HDSS: health and demographic surveillance system
HMIS: health management information system
ICD: international classification of disease
ICER: incremental cost-effectiveness ratio
ITT: intention-to-treat
MDGs: millennium development goals
PCV 10: 10-valent pneumococcal conjugate vaccine
PCV 13: 13-valent pneumococcal conjugate vaccine
TBA: traditional birth attendant
TTBA: trained traditional birth attendant
US$: united states dollar
VA: verbal autopsy
WHO: world health organization
