# Supplementary material for: Cost-effectiveness of childhood pneumococcal vaccination program in Ethiopia: results from a quasi-experimental evaluation
Source: BMC Public Health. 2019 Aug 9;19:1078. doi: 10.1186/s12889-019-7423-8 (PMC6688319; doi:10.1186/s12889-019-7423-8)
Supplement: Supplementary file 1 — Household survey questionnaire. (DOCX 17 kb) [file 12889_2019_7423_MOESM1_ESM.docx]

## Questioner (English)

Questionnaire for data collection on treatment cost of childhood pneumococcal infections at Butajira rural health program site, 2015

**Verbal Consent Form before Conducting the Interview**

Hello, how are you?

My name is ---------------------------------------. I am working in the research team that works to determine cost effectiveness of pneumococcal vaccination at Butajira rural health program site. I would like to interview you few questions about your expenses that you have incurred to get treatment for your child’s recent pneumococcal infection. The objective of the study is to measure the cost-effectiveness of PCV 10 vaccination in the program site. Your cooperation and willingness for the interview is very helpful in identifying the problems related to the issue. Your name will not be written in the form and I assure you that all information that you give will be kept strictly confidential. Your participation is voluntary and you are not obliged to answer any question you do not wish to answer. If you are not still comfortable with the interview, please feel free to stop it any time you like. Do I have your permission to continue?

1 – If yes, continue to the next page

2 – In no, skip to the other participant

**Thank you!!**

Interviewer’s name and code-------------------------------------------------, signature------

Date if interview------------------------, Time started _______________, Time finished ------------

Supervisor’s name ----------------------, Signature --------------------------

Respondent ID_______________

Questions

1. In the last three years, do you remember how many times get your child pneumococcal infection?
2. Yes B. No

If yes, how many times________________

1. How often did you take your child to hospital/ health center when s/he got pneumococcal infection? ________________
2. When was the last time that you took your child to hospital/health center when s/he got pneumococcal infection? ____________________
3. What was your means of transportation for your child’s recent health care visit (for the above-mentioned case) and how much did you pay for each means?
4. Public transport __________________
5. Private car _____________________
6. Traditional stretcher ________________
7. Animal transportation ________________
8. Walk
9. Other (specify___________________)
10. Mode of treatment for your child’s recent health care visit (for the above-mentioned case)
11. Inpatient
12. Outpatient
13. If outpatient, how long did you visit the hospital/health center? ____________
14. If inpatient, for how long did you stay at the hospital/health center? _______________
15. Was there anybody accompanied you when you took your child to the hospital/health center? A. Yes B. No
    1. If yes, who was s/he?
    2. Was s/he absent from work due to your child’s infection? A. Yes B. No
    3. How long was s/he absent from work?
    4. How much s/he earns per month? (on average) _______________________
16. For how long was you absent from your work due to your child’s recent infection? __________________________________
    1. How much earns you per month (on average) _________________
17. Was there anybody absent from wok to took care of your child at home when s/he was sick other than you? A. Yes B. No
    1. If yes, how long s/he was absent from work? _____________
    2. If yes, how much s/he earns per month ______________
18. For how long need your child personal assistance when s/he was sick in the recent episode? _______________________
19. How much did you pay (for the above-mentioned case)?
    1. For card _______________
    2. For diagnosis _________________
    3. For inpatient care __________________
    4. For drugs _________________
    5. For food _________________
    6. For accommodation ____________
    7. Other (specify) ____________________
20. Has your child fully recovered? A. yes B. no
    1. If no, what measures did you take?
21. ________________________________ how much did you pay? ___________
22. ________________________________ how much did you pay? ___________
23. ________________________________ how much did you pay? ___________
    1. If no, what is your future plan?
